# The NANOS RNA-binding protein variants: a model for understanding human infertility

**DOI:** 10.1007/s13353-025-01009-8

**Published:** 2025-10-18

**Authors:** Amanda Kunik, Bellary Lakshmi, Jadwiga Jaruzelska, Kamila Kusz-Zamelczyk

**Affiliations:** Institute of Human Genetics, Polish Academy of Sciences, Strzeszynska 32, 60-479 Poznan, Poland

**Keywords:** Human infertility, NANOS variants, Germ cell homeostasis

## Abstract

Infertility remains a significant global health challenge. With up to one in seven couples affected worldwide, the inability to conceive has become a major concern for reproductive health. Many causes of infertility are linked to genetic variants that are disruptive for germ cell homeostasis. Using causative variants to model human infertility can improve our understanding of the molecular pathways that regulate germ cell development. One notable example is the germ cell morphogen NANOS, which is highly conserved across species. Variants of NANOS are associated with infertility in various animal models, from *Drosophila* to humans. Here, we examine how modelling human infertility based on NANOS variants can offer insights into the molecular processes underlying germ cell development. Ultimately, uncovering the molecular basis of human infertility through this approach is vital for developing advanced diagnostic methods and therapeutic options in the future.

## Introduction

Infertility is a significant global health issue. The inability to conceive has become a major concern for human reproductive health, affecting up to one in seven couples worldwide; for a review, see Sang et al. (2023). Couples who cannot conceive often choose in vitro fertilisation. However, while this procedure represents a breakthrough in addressing infertility, it is only successful for a limited percentage of couples. Therefore, the question arises of how to improve the diagnosis and treatment of infertility.

Since many causes of infertility are linked to variants of genes that are essential for germ cell homeostasis, examining such variants can provide insights into the mechanisms necessary for different stages of germ cell development. When disrupted, these mechanisms can lead to infertility in humans. Specifically, depending on their expression time, variants may be useful for modelling human infertility at various stages of germ cell development, including germ cell specification, migration to the primary gonad, or gametogenesis. Such modelling could enhance our understanding of the molecular pathways affected by mutations, which are important in germ cell specification and development. Ultimately, understanding the molecular pathways underlying human infertility is crucial for developing innovative diagnostic tools and treatments.

Whole-genome sequencing technologies have identified variants in many genes encoding proteins linked to human infertility. When selecting protein-coding gene variants associated with infertility to explore the fundamental mechanisms of human reproduction, the main criteria are as follows: a proven causative effect, evolutionary conservation of the gene, including its essential role for reproduction in other species, and the stability of the protein variant. One notable example of such a gene is *NANOS*, which is highly conserved across animal species. *NANOS* plays a vital role in germ cell development, as demonstrated in different animal models from *Drosophila* to mammals, and stable protein variants of NANOS have been proven to cause human infertility.

## NANOS is a highly conserved germ cell morphogen

NANOS is an extensively studied morphogen involved in germ cell development, found in organisms ranging from desmosponges to humans. It was first described in *Drosophila* as a unique gene with no paralogues, crucial for body patterning and germ cell maintenance, which hinders their somatic fate (Asaoka-Taguchi et al. 1999). However, during evolution, more paralogues emerged, resulting in three paralogues in mammals: NANOS1, NANOS2, and NANOS3. Each of the NANOS paralogues has distinct reproductive functions. Specifically, while NANOS2 is involved in male germ cell development, NANOS1 and NANOS3 are expressed much earlier, before primordial germ cells (PGCs) are sexually determined, and are expected to be important for germ cell development in both sexes (Sybirna et al. 2020).

**Fig. 1:**
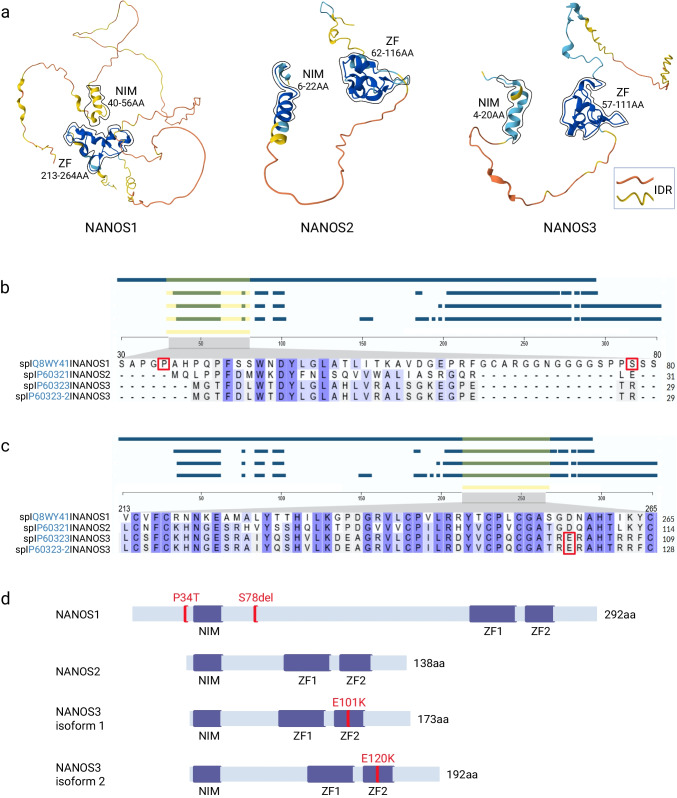
Human NANOS paralogues. **a** Alpha Fold structural homology models of NANOS1 (AF-Q8WY41-F1-v4), NANOS2 (AF-P60321-F1-v4), and NANOS3 isoform 1 (AF-P60323-F1-v4) proteins, derived from the EMBL-EBI Alpha Fold protein structure database. These structures include the conserved NIM region at the N-terminus and the ZF domain 2xCCHC at the C-terminus. The NIM region and ZF domain of each NANOS paralog are enclosed by thin lines. A major part of these structures is IDR, which indicates varying degrees of structural flexibility. The pLDDT values, ranging from very high to very low confidence, are colour-coded: dark blue (very high confidence, pLDDT > 90), light blue (high confidence, 90 > pLDDT > 70), yellow (low confidence, 70 > pLDDT > 50), and orange (very low confidence, pLDDT < 50). **b**, **c** Multiple sequence alignment of the NANOS1, NANOS2, and both isoforms of NANOS3 proteins was carried out using the Clustal Omega program (CLUSTAL O 1.2.4). The yellow-highlighted area from each alignment is further enlarged (grey) to display the sequence similarity within this region. Amino acid positions that are susceptible to mutations are marked in red boxes. **d** Schematic representation of NANOS1, NANOS2, and both isoforms of NANOS3. Mutations that have been proven to cause infertility are indicated and marked in red. Created in BioRender: https://BioRender.com/sxbxls7. Abbreviations: *NIM*, NOT1-interacting motif; *IDR*, intrinsically disordered region; *pLDDT*, predicted local distance difference test; *ZF*, zinc finger domain

NANOS is a post-transcriptional regulator that controls the level of specific mRNAs in germ cells. It is an RNA-binding protein with a conserved C-terminal RNA-binding domain composed of two zinc fingers (2xCCHC) that interact with RNAs and proteins (Fig. 1). The N-terminal region is unstructured and in vertebrates contains a short segment known as the NOT-interacting motif (NIM). This motif recruits the deadenylation complex by direct binding to the CCR4-NOT transcription complex subunit 1 (CNOT1) scaffold protein, promoting the deadenylation and degradation of mRNA targets (Fig. 1) (Bhandari et al. 2014). NANOS’ best-known protein partner is PUMILIO, as established in *Drosophila* (Asaoka-Taguchi et al. 1999) and later in humans (Jaruzelska et al. 2003). PUMILIO, a well-studied RNA-binding protein, recognises a specific 8 nucleotide UGUAHAUA motif usually located in the 3′UTR of mRNA targets across various organisms, including humans (Bohn et al. 2018). By binding to the 3′UTR and depending on the set of auxiliary proteins attached, mRNA targets undergo deadenylation and degradation or storage within membraneless cytoplasmic structures called P-bodies. The mRNA targets may also be directed to other regions of the cytoplasm, depending on motifs in the 3′UTR, which is vital for their function. While NANOS operates as an effector for PUMILIO mainly by promoting the degradation of PUMILIO-bound targets (Bohn et al. 2018), it also represses mRNA targets independently of PUMILIO, as demonstrated for NANOS2-bound mRNAs in mouse spermatogonia (Codino et al. 2021). This raises the question of whether NANOS2 requires additional proteins to select the correct RNA target for repression in these cells. Indeed, the RNA-binding protein Dead End Protein Homolog 1 (DND1) has been shown to interact and cooperate with NANOS2 to load mRNA targets onto the deadenylation complex for deadenylation, a process vital for mouse germ cell development (Suzuki et al. 2016). NANOS2’s zinc-finger domain mediates interaction with DND1, which is essential for its binding to target RNAs. The next question is, what is the fate of the NANOS2-repressed mRNA targets? Importantly, NANOS2 promotes the localisation of CNOT proteins to P-bodies in vivo (Suzuki et al. 2016). An important question is whether such protein–protein interactions of NANOS2 are germ cell stage-specific.

## NANOS, a component of germ granules that control germ cells in the animal kingdom

The germ granules, a key feature of germ cells, are vital for their specification and development in the animal kingdom. These cytoplasmic, membraneless structures form condensates containing RNAs and RNA-binding proteins, creating ribonucleoprotein complexes that undergo dynamic remodelling at specific stages of female and male germ cell development. In germ granules of *Drosophila* oocytes, NANOS mRNA molecules are translated in later stages of germ cell development, as reviewed (Lakshmi et al. 2025). Depending on the species, germ granules form a higher-order structure called germ plasm, which is inherited from the oocyte. As observed in lower organisms, cells inheriting the germ plasm develop into germ cells. In contrast, in mammals, germ granules are not maternally inherited, and germ plasm is absent. Mammalian germ cells specify from a group of proximal epiblast cells in response to external signals from surrounding extraembryonic tissues during early stages of embryogenesis. In humans, germ granules were first identified in primordial germ cells after they entered the primary gonads (Kellokumpu-Lehtinen and Soderstrom 1978). However, the precise timing of human germ cell specification, including germ granule formation, has not been determined in vivo due to ethical concerns. Recent advances in understanding these processes have been made through the development of in vitro models for germ cell specification and early development from human pluripotent stem cells, such as the W15 cell line (Irie et al. 2015). This raises an important question: do germ granules form earlier than previously believed, for example, before migration and reaching the primary gonads? If so, what are the dynamics of their formation? Based on this in vitro model, it was found that NANOS3 and, more recently, NANOS1 begin to be expressed during PGC specification (Irie et al. 2015; Sybirna et al. 2020). Although NANOS3 is considered a marker of mammalian germ cell specification, including in humans, its role at this early stage of germ cell development has never been defined. Furthermore, the role of NANOS1 at this stage remains unknown. Therefore, an open question remains: do germ granules begin to form at this stage, and if so, are NANOS1 and NANOS3 involved in that process?

Germ granules serve as centres for RNA processing and post-transcriptional regulation during germ cell development. Consequently, NANOS, a posttranscriptional regulator of gene expression that binds RNA and is present in germ granules across various organisms, seems to be an essential component of their structure.

Below, we explore the role of individual NANOS paralogues in human infertility and consider the potential of NANOS variants for modelling human infertility to investigate the fundamental processes of germ cell development. Specifically, the identification of RNA targets and protein interactions disrupted in NANOS infertility-linked variants seems to be a way to identify pathways critical for germ cell development. Does this impairment affect germ granule formation and structure? If so, how do NANOS paralogues regulate these processes? We believe that using NANOS variants to create a human infertility model could help address these questions and deepen our understanding of the molecular mechanisms involved.

## NANOS2 gene and male infertility

The NANOS2 gene plays a critical role in male germ cell development in mice immediately after PGCs enter the primary gonads. Later, NANOS2 contributes to the maintenance of spermatogonia by counteracting their differentiation. Disruption of NANOS2 results in male infertility (Tsuda et al. 2003). Therefore, a study was conducted to investigate the NANOS2 gene for variants in a group of 214 infertile male patients with non-obstructive azoospermia or oligozoospermia from the Polish cohort, alongside 400 fertile males from the same population. The study identified two heterozygous variants in two different oligospermic patients. The effect of the first variant p.H68Q, on sterility was uncertain, as it co-occurred with a microdeletion in the Azoospermia factor (AZF) region of the Y chromosome. The second variant was a silent single-nucleotide substitution, p.H109H. Although both variants were located within the most conserved RNA-binding domain and absent in the 400 fertile males, it remains unclear whether they contribute to male infertility, partly due to limited data on other family members (Kusz et al. 2009b).

## NANOS3 gene in male and female infertility

In mice, NANOS3 expression begins much earlier than that of NANOS2—at the time of PGC specification, well before migration to the gonadal ridges (Sybirna et al. 2020). Accordingly, knocking out the NANOS3 gene in mice results in the complete loss of germ cells in both sexes (Tsuda et al. 2003). The NANOS3 gene is expressed from the early stages of PGC formation and is therefore considered a marker of PGCs (Sybirna et al. 2020) and continues into adulthood in gonadal germ cells. It is known to protect PGCs from apoptosis during migration, as demonstrated in mice, and is essential for gametogenesis (Tsuda et al. 2003). The role of NANOS3 in the early stages of PGC formation is not yet understood.

The significance of NANOS3 in human fertility is well-established, particularly concerning women’s reproductive health. Specifically, the NANOS3 homozygous gene variant p.E120K, linked to premature ovarian insufficiency, was identified in two Brazilian sisters and produced a stable NANOS3 protein (Table 1). Notably, this amino acid substitution was located within the zinc finger domain. It was expected that disrupting this highly conserved region could impair NANOS3’s ability to bind mRNA targets or protein partners. Additionally, in vitro studies indicated that COS-1 cells carrying this NANOS3 variant showed reduced resistance to apoptosis compared to wild-type cells (Santos et al. 2014). It was also demonstrated that NANOS3 directly binds the 3′UTR of mRNA encoding Siah E3 Ubiquitin Protein Ligase 1 (SIAH1), along with PUMILIO2, and represses it. Importantly, the NANOS3 protein with the p.E120K variant could not repress *SIAH1* mRNA as effectively as the wild-type NANOS3 (Sajek et al. 2019). However, a comprehensive search of germ cells for targets bound by this NANOS3 variant, compared to the wild-type, has not been reported.

**Table 1:** Experimentally validated NANOS protein variants associated with infertility

Type of NANOS	Variant ID (GnomAD)	Transcript	Amino acid alteration	Disease association	References
NANOS1	10-119029901-C-A	ENST00000425699.3	p.P34T	Heterozygous double variant (both p.P34T and p.S78del in one allele) p.[P34T;S78del] associated with *Sertoli cell only syndrome* (SCOS)	Kusz-Zamelczyk et al. 2013﻿
	10-119030030-CCCT-C		p.S78del		
NANOS3	19-13877606-G-A	ENST00000397555.3 (isoform 1)	p.E101K	Homozygous variant p.E101K/p.E120K associated with *premature ovarian insufficiency* (POI)	Santos et al. 2014
		ENST00000339133.6 (isoform 2)	p.E120K		

The NANOS3 variants may potentially not only cause fertility issues in women but also impact men. Therefore, screening for NANOS3 gene variants was conducted on a group of 214 patients from the Polish cohort diagnosed with non-obstructive infertility, including azoospermia or oligospermia. The analyses found no potentially pathogenic variant in NANOS3 within this group (Kusz et al. 2009a).

## NANOS1 gene in male infertility

While disruption of NANOS2 and NANOS3 in mice causes infertility, NANOS1, although expressed in germ cells, does not seem to play a significant role in reproduction or other processes in mouse physiology (Haraguchi et al. 2003). Surprisingly, this does not appear to be the case for human NANOS1. Specifically, to determine whether NANOS1 plays a crucial role in human fertility, researchers examined the same group of infertile males for non-obstructive azoospermia or oligozoospermia, as described (Kusz et al. 2009a, 2009b) for variants of the NANOS1 gene. They identified a heterozygous variant allele with a double mutation p.[P34T; S78del] in two patients with azoospermia and a complete absence of germ cells in the seminiferous tubules, based on biopsy analysis, which is known as Sertoli cell-only syndrome (Kusz-Zamelczyk et al. 2013) (Table 1). This variant was not identified in the cohort of fertile males from Polish or other general populations that were screened. Conversely, women heterozygous for this variant remained fertile, as demonstrated in the family pedigrees carrying the p.[P34T; S78del] variant (Kusz-Zamelczyk et al. 2013). To validate the significance of the p.[P34T; S78del] variant in human reproduction, its potential role was tested in the TCam-2 cell line, which models human PGCs at the time they enter the primary gonad. Overexpression of the variant protein induced apoptosis, whereas wild-type NANOS1 exhibited an anti-apoptotic function (Janecki et al. 2020). Additionally, since NANOS1 is an RNA-binding protein (RBP), RNA-seq data were analysed to identify targets encoding genes involved in apoptosis. Notably, overexpression of wild-type NANOS1 resulted in the downregulation of several pro-apoptotic genes, including *GADD45A*, *GADD45B*, *GADD45G*, and *RHOB*, whereas this was not observed with the variant NANOS1 in the same cell line. Interestingly, these targets encode proteins with closely related functions—factors that activate DNA damage-induced apoptosis (Janecki et al. 2020). This effect supports the phenotype observed in patients lacking germ cells in the seminiferous tubules. Interestingly, the variant encompasses the NIM region, which, as mentioned earlier, is responsible for recruiting the deadenylase complex for target mRNA deadenylation and degradation (Fig. 1) (Bhandari et al. 2014; Kusz-Zamelczyk et al. 2013). It would be of interest to determine whether this variant affects the recruitment of the deadenylase complex. Moreover, the p.S78del of that variant is located in a 25-amino-acid N-terminal region, which is absent in mice (Kusz-Zamelczyk et al. 2013). This suggests that a gain-of-function mutation in NANOS1 may have occurred during evolution, making NANOS1 essential for human fertility, in contrast to mice.

In the aforementioned previous studies, NANOS3 interacted with PUMILIO2 to downregulate *SIAH1* mRNA, while the variant was significantly less efficient (Sajek et al. 2019). Also, the NANOS1 variant was further investigated in this context. This study revealed that NANOS1 exerted a repressive effect on PUMILIO2-bound *SIAH1* mRNA, whereas the NANOS1 variant-induced suppression of *SIAH1* mRNA was significantly less effective when transiently expressed in HEK293 cells. However, the significance of that repression for germ cell homeostasis was not clarified (Sajek et al. 2019).

The cellular sub-localisation of NANOS1 during spermatogenesis revealed it to be cytoplasmic perinuclear (Jaruzelska et al. 2003). In later stages of spermatogenesis, such as in round spermatids, NANOS1 was found in the chromatoid body (CB), a mammalian spermatogenesis-specific, singular cytoplasmic round structure that interacts with the cell nucleus in germ cells, including humans. The CB is a type of germ granule that serves as an RNA processing centre; for a review, see (Lakshmi et al. 2025). The CB was identified in the round spermatids of mice and humans using DEAD-Box Helicase 4 (DDX4) protein staining, which serves as a marker for the CB, as shown in the mouse (Ginter-Matuszewska et al. 2011). It was discovered that the human CB contains NANOS1, PUMILIO2, and DEAD-Box Helicase 20 (DDX20) proteins, all of which interact with each other (Ginter-Matuszewska et al. 2011). Interestingly, interaction between NANOS1 and DDX20 was significantly weakened in the presence of NANOS1 variant (Kusz-Zamelczyk et al. 2013). Therefore, the question arises as to whether such a variant induces remodelling of CB and, if so, whether such remodelling contributes to infertility caused by that NANOS1 variant. Thus far, NANOS1 is the only component of germ granules for which a variant causing human infertility has been identified.

The question is whether NANOS1 is involved in germ granule formation, as its *Drosophila* homologue is. Although germ granules are described in human PGCs at the stage when they reach the gonad, the inaccessibility of early human PGCs has hindered further studies on the beginning of germ granule formation.

## Future perspectives of using NANOS-based model for understanding human infertility

**Fig. 2:**
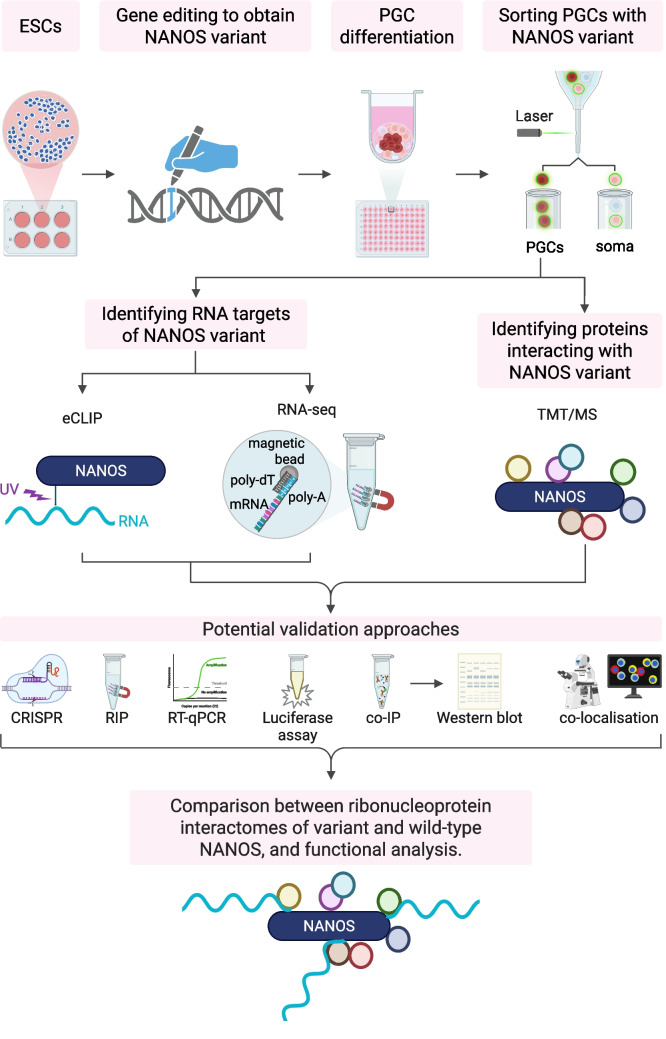
Understanding variant NANOS-associated molecular pathways underlying human infertility. A schematic overview of a plausible in vitro approach to learn about the RNA/protein interactome of variant NANOS, aiming to understand the developmental anomalies that occur, especially during primordial germ cell formation. In this scheme, ESCs serve as a model system, which are differentiated into PGCs, and sorted and analysed to identify variant NANOS-specific RNA targets using eCLIP and RNA-seq. The interacting proteins can be identified using TMT/MS. Created in BioRender: https://BioRender.com/n1xjtfi. eCLIP, enhanced cross-linking and immunoprecipitation; ESCs, embryonic stem cells; PGCs, primordial germ cells; MS, mass spectrometry; TMT, tandem mass tag

Several important questions remain to be answered, particularly when modelling human infertility based on infertility-associated NANOS variants. Since NANOS proteins interact with both RNA and proteins, a comprehensive search for RNA targets can be carried out using CLIP- or TRIBE-based methods alongside transcriptomic studies, as well as investigations of auxiliary proteins through proteomic approaches. This will help identify the ribonucleoprotein interactome of wild-type and variant NANOS1 and NANOS3, as presented in Fig. 2. The next step is to determine whether these interactions are disrupted and if potential phenotypic effects occur during the differentiation of germ cells carrying NANOS infertility-related variants. Additionally, since NANOS1 binds to the 3′UTR regions of target mRNAs, which contain localisation signals, it is expected that the variant may cause RNA target mislocalisation. However, due to ethical considerations, access to early in vivo stages of PGC specification and development, as well as the use of these cells for modelling, is limited. Therefore, in vitro models involving the differentiation of human pluripotent stem cells into PGCs, such as the W15 pluripotent stem cell line, are highly valuable (Irie et al. 2015). Pluripotent stem cell lines can be genetically modified to express either mutated NANOS1 or NANOS3 and then differentiated into PGCs, after which their phenotype can be analysed and compared. A phenotypic effect in the cell lines expressing the variant NANOS1 or NANOS3 protein that aligns with the patient’s phenotype would be of value and would validate the model. Finally, such a model can be used to identify disrupted molecular pathways underlying infertility caused by the variants, providing advanced diagnostic methods and therapeutic options in the future.
